# Amalgam and Oxyphosphates as Filling-Material

**Published:** 1899-01

**Authors:** C. W. Strang

**Affiliations:** Bridgeport, Conn.


### AMALGAM AND OXYPHOSPHATES AS FILLING-MATERIAL.[1]

BY DR. C. W. STRANG, BRIDGEPORT, CONN.

INASMUCH as gold as a filling-material is excluded from deciduous teeth, inasmuch as we are all agreed that gold is not a fit material to use in immature and undeveloped permanent teeth, inasmuch as we have a large proportion of cases of persons who have inherited a fairly good set of teeth and by a systematic and judicious course, both upon their dentist's and their own part, have preserved them oftentimes until the threescore limit and even later, and then for some unknown cause or reason we are astounded on some occasions to find them rapidly failing, softening at the margins, fillings that have perhaps done duty for fifteen, twenty, or thirty years giving way. Inasmuch as we have many applying to us for treatment who have but little power of endurance, there is, you see, a large field where we must look for some other material than gold as a filling-material. I would not advocate any system of practice that would lower the standard of dentistry, but the question that we are called upon to solve is not what can be done, but what ought to be done.

The questions which come before us almost every day of our lives are, How may I permanently preserve the teeth of my patients? How may I maintain a high degree of excellence in my operation without inflicting unnecessary pain and suffering? Now, if gold must necessarily be excluded from a number of the operations that we perform, then the next question that comes to us is, What will take the place of gold? When I started in dentistry, over thirty years ago, we were chiefly confined to tin and gold as filling-materials. Tin preserved remarkably well in those cases which were not subjected to much stress or strain. But as years passed by, somehow or other, tin began to pass out of use. Those who had been taught to use tin passed over to amalgam, until, at the present time, we will hardly find a dentist in practice who resorts to tin as a filling. This, I believe can be attributed to the ease with which amalgam filling is inserted, and the care that must necessarily be used if we employ tin.

About ten years ago, the use of a combination of amalgam and oxyphosphate was suggested to me. I immediately began to experi-

---

[1] Read before the Northeastern Dental Association, Hartford, Conn., October 19, 1898.

ment in mixing amalgam and oxyphosphate. More and more I began to adopt this preparation for a filling-material in cases where gold should not be used. For instance, take the deciduous teeth. I think most of us are apt to underestimate the stress brought upon the molars. I have never seen a good amalgam filling in deciduous teeth that was a year or a year and a half old. Very often the naked eye can find fissures in the filling, or the amalgam may be broken at the edges. So far as my observation and experience go, there is nothing better to preserve deciduous teeth than the filling composed of amalgam and oxyphosphate of zinc. Using this material we may cut away from the grinding surface as much as we desire. But if this material is inserted much care must be taken to exclude all moisture from the cavity. Unless your cavity is dry, the filling will be worthless. But if you are able to get the cavity dry you may put in this filling with the assurance that, when you look at it a year hence, you will find it absolutely perfect at the margins.

Now, again, in regard to the care of the permanent teeth,—the unsolidified permanent teeth. We frequently find defects in the incisors as early as the tenth or twelfth year. I want to say that I regard filling the anterior teeth with gold prior to the age of eighteen or twenty as a species of malpractice. I do not presume to advise those men as old or older than myself, but I want to say to the young men, Be not hasty in introducing gold into the undeveloped, unsolidified permanent teeth. If I could take you to my own office, I could show you teeth that have been carrying pink gutta-percha fillings for over ten years, and they are perfectly preserved and in as good condition as they were the day they were filled. I use pink gutta-percha in anterior teeth, because I have found that it is more enduring. Of course, if the cavity is conspicuous, we must adopt the white.

About seven or eight years ago, a lady came to my office bringing her daughter, a girl of about thirteen or fourteen years of age. There were present in the girl's front teeth, or there had been in her incisors, five or six gold fillings. I found one gold filling tight in the cavity, one loose, and the others had disappeared. I asked the lady if she had insisted upon having gold inserted as a filling. She said that the dentist had done as he pleased. He had advised gold and used it. I told the lady that I should not advise gold, but would fill the teeth with gutta-percha and guarantee that, with occasional repairing, they would be as good five years hence as they were that day. So it proved, and the cavities are precisely the same now as they were at least eight years ago when the operation was performed. This is simply a record of the uniform result of such cases. But when it comes to approximal cavities in molars and bicuspids, we frequently find that when the separator has been applied it would be impossible to introduce even gutta-percha without considerable excavation. For a number of years it has been my practice to get a little groove or, perhaps, two or three retaining pits and apply the amalgam cement. I have been surprised to find how remarkably these fillings preserve the teeth.

Take another class of teeth, teeth which by decay have become very weak. If we fill these teeth with gold, the enamel walls are so frail that it is necessary for us to remove about one-third or one-fourth of the crown, and to build a bevelled enamel wall means three or four hours of hard work. And it means a great strain upon your patient. Many are unable to endure this strain even if they are able to pay for the work. I have in my own practice been better satisfied with a fair removal of weak enamel walls and the use of the enamel cement. I advise the patient not to be reckless in the use of his teeth because they are weak. They may, with care, do good service, but are liable to break off. This method has been more satisfactory than to resort to the more heroic method of levelling off the walls and restoring with gold. From the experience I have had with the use of this preparation, I find it makes no difference what amalgam you use. Some amalgams make a very dark, some a fairly light filling, others a more homogeneous color. Some amalgams are preferable on account of the color. It makes a great difference as to what oxyphosphate is used. All quick-setting oxyphosphates must be excluded. It also makes a difference as to the dexterity with which you use it. Do not mix it expecting to fill two cavities with the same kind. When the preparation has been properly combined, you must insert it in the cavity without an instant's delay. Every instrument needed for the operation must be at hand, so that no time be lost. Unless this is done there will be danger of disintegration. My method has been, whatever amalgam I use, to put it into a mortar and add enough of mercury to make a mass so that it will not crumble if pressed between the fingers. When this is thoroughly mixed I use about one-third in bulk of the powder. Mixing this all together will give you a black mass. I then place on the tablet sufficient liquid to properly mix up the oxyphosphate powder used. When you get this into a mass similar to putty, it is ready for use, and should be put into the cavity as expeditiously as possible and pressure be brought upon it. If an approximal cavity, use a matrix in order that pressure be made upon the mass. In the course of two or three minutes you can remove the band and trim to required shape. If the mass is of the proper consistency, it adheres to the cavity just as enamel does to the teeth, providing the cavity be kept dry.

I am not particular about getting in any undercuts. Generally the shape of the cavity is enough. The filling adheres so closely to the walls of the cavity that there is little danger of it being displaced. I do not recall a single instance in which the filling has been disturbed. The use of this material requires care. The cavity must be thoroughly prepared, just as thoroughly as for a gold filling. The instruments must be kept clean. Your slab must be kept clean. Anything foreign brought into this mass will cause it to disintegrate, and it would be better to throw it away.

